# Supplementary material for: Apelin modulates inflammation and leukocyte recruitment in experimental autoimmune encephalomyelitis
Source: Nat Commun. 2024 Jul 25;15:6282. doi: 10.1038/s41467-024-50540-5 (PMC11282314; doi:10.1038/s41467-024-50540-5)
Supplement: Supplementary file 1 — Supplementary Information [file 41467_2024_50540_MOESM1_ESM.pdf]

Apelin modulates inflammation and leukocyte recruitment in experimental autoimmune encephalomyelitis

Hongryeol Park[1], Jian Song[2], Hyun-Woo Jeong[1], Max L. B. Grönloh[3], Bong Ihn Koh[1], Esther Bovay[1], Kee-Pyo Kim[4], Luisa Klotz[5], Patricia A. Thistlethwaite[6], Jaap D. van Buul[3], Lydia Sorokin[2], Ralf H. Adams[1,#]

Table of contents

Supplementary Figures. 1-22

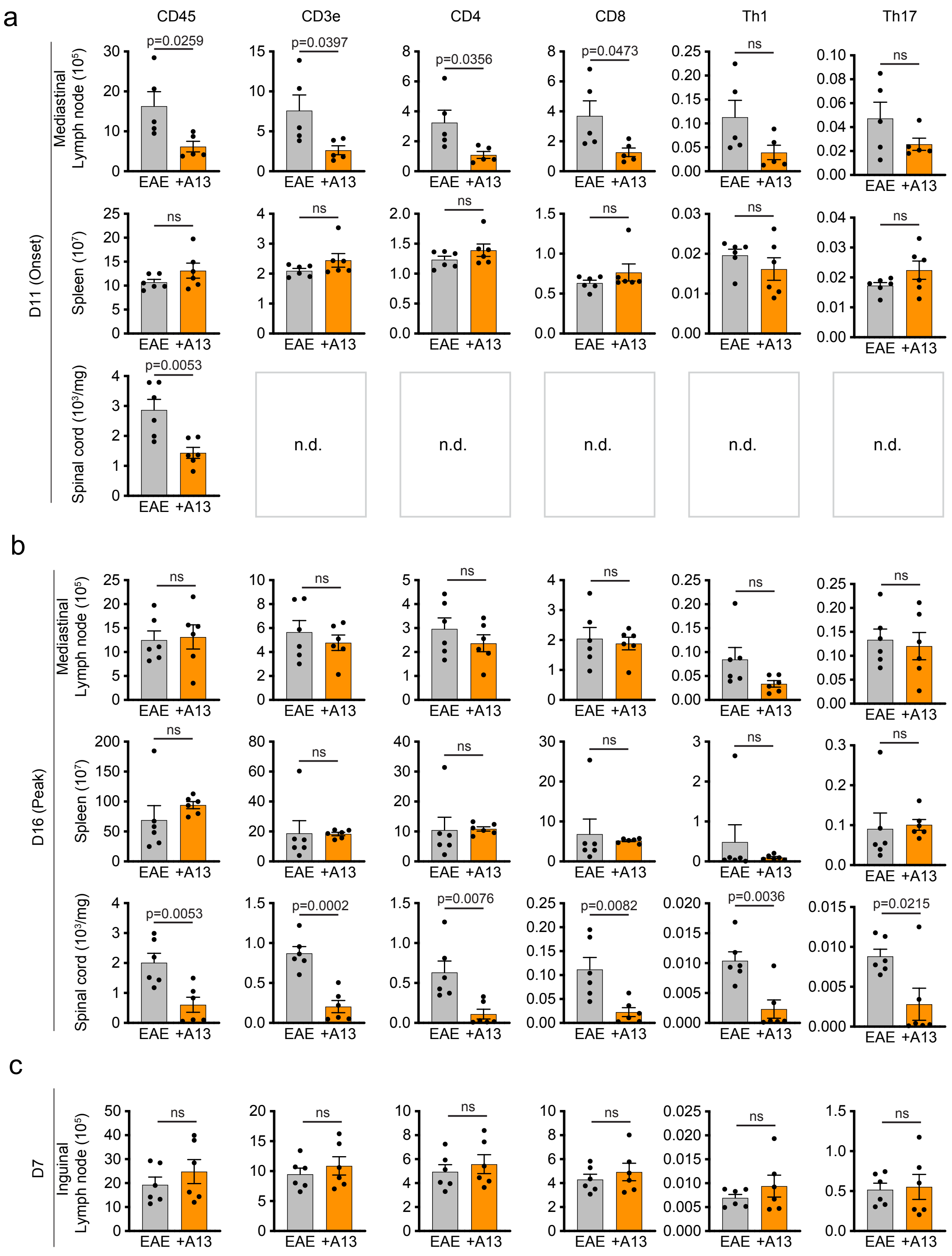

Supplementary Figure 1. Effect of A13 on lymph nodes, spleen and spinal cord.

a, b, Number of the indicated immune cell populations in mediastinal lymph node, spleen, and spinal cord from EAE +vehicle (grey boxes) and +A13 (orange boxes) mice at D11 (a) and D16 (b). T cell populations were below detection limit ("n.d.") in spinal cord at D11. Error bars, s.e.m. p-value, student t-test (*$p<0.05$, **$p<0.01$, ns, not statistically significant). c, Number of immune cells in inguinal lymph nodes from EAE +vehicle (grey boxes) and +A13 (orange boxes) mice at D7. Error bars, s.e.m. Student t-test (ns, not statistically significant). The number of mice is 6 for each group, except for the D11 Mediastinal Lymph node group, which has 5 mice each.

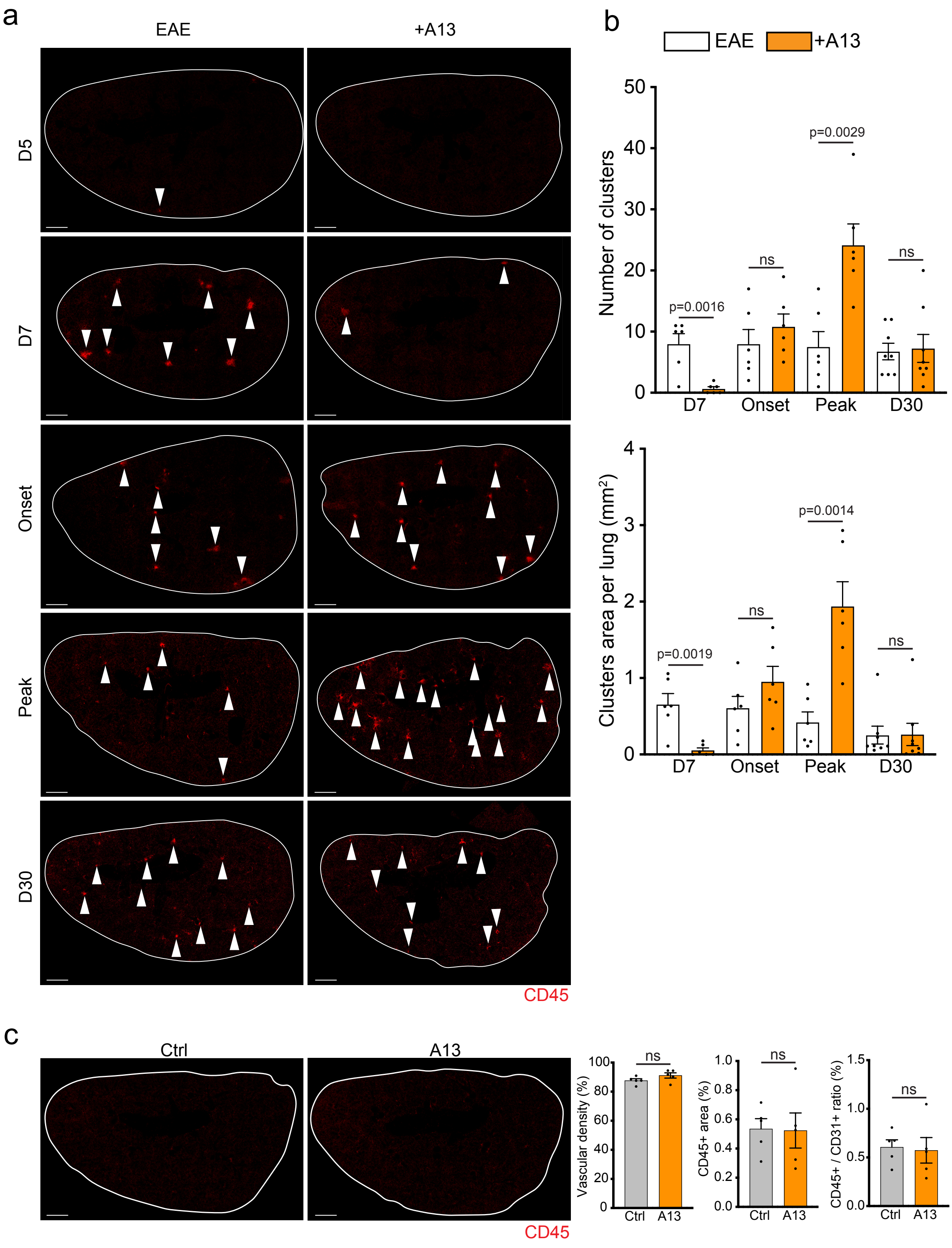

Supplementary Figure 2. CD45+ cell accumulation in EAE lung.

a, CD45+ immune cell clusters (arrowheads) in lung lobe sections (white outline) at the indicated stages after EAE induction. Note that A13 delays immune cell accumulation but, at peak EAE, also the resolution of CD45+ clusters. Scale bar, 1mm. b, Quantification of number of CD45+ immune cell clusters and clusters per area in lung sections. Error bars, s.e.m. Student t-test (ns, not statistically significant). The number of mice is 6 for each group, except for the D30 group, which has 8 mice each. c, Absence of CD45+ cell clusters in the lung of naïve mice treated with A13 for 14 days. Graphs on the right show that vascular density, CD45+ area and ratio of CD45+ and CD31+ areas are unaffected by A13. Error bars, s.e.m. Student t-test (ns, not statistically significant).). The number of mice is 5 for each group.

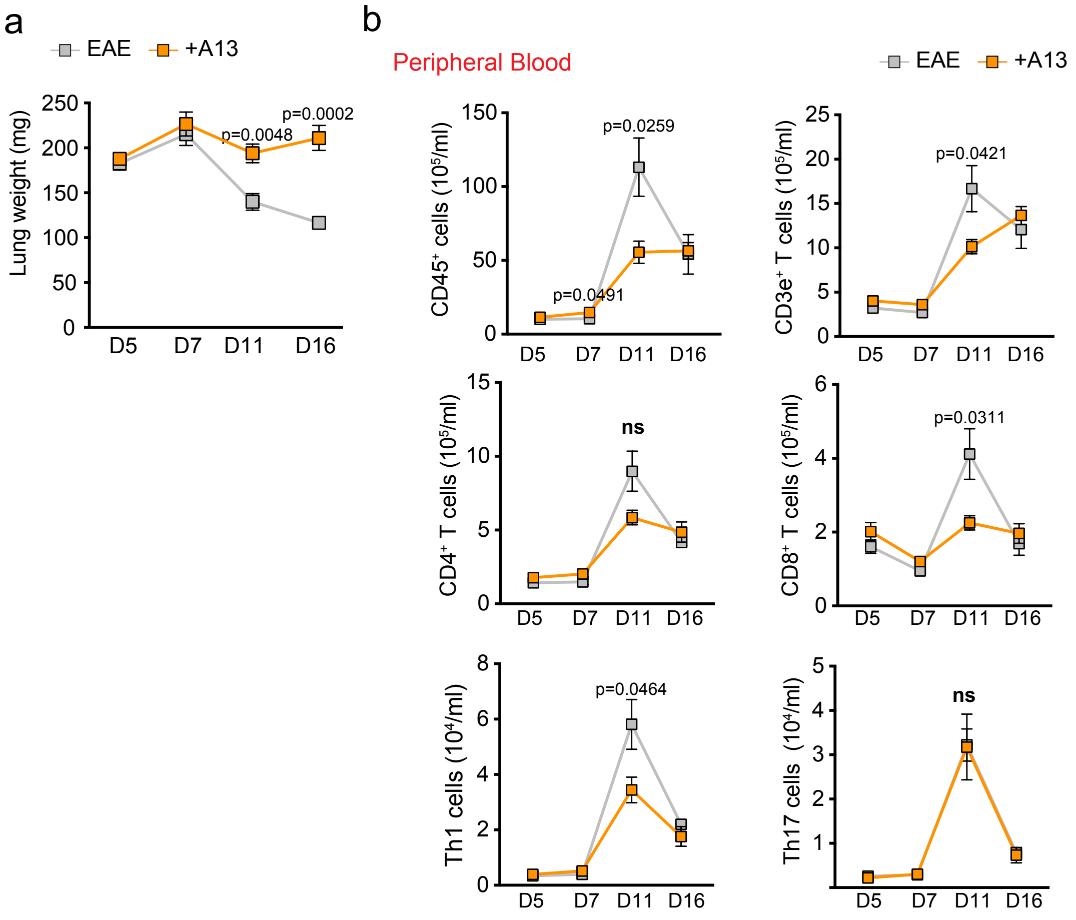

Supplementary Figure 3. Effect of A13 on lung weight and circulating immune cells.

a, Lung weight during EAE progression (grey line) and impact of A13 treatment (orange line). Error bars, s.e.m. Student t-test. The number of mice is 5 for each group. b, Number of immune cell populations in EAE (grey lines) and A13-treated (orange) peripheral blood at the indicated days after immunization. Error bars, s.e.m. Student t-test (ns, not statistically significant). The number of mice is 5 for each group.

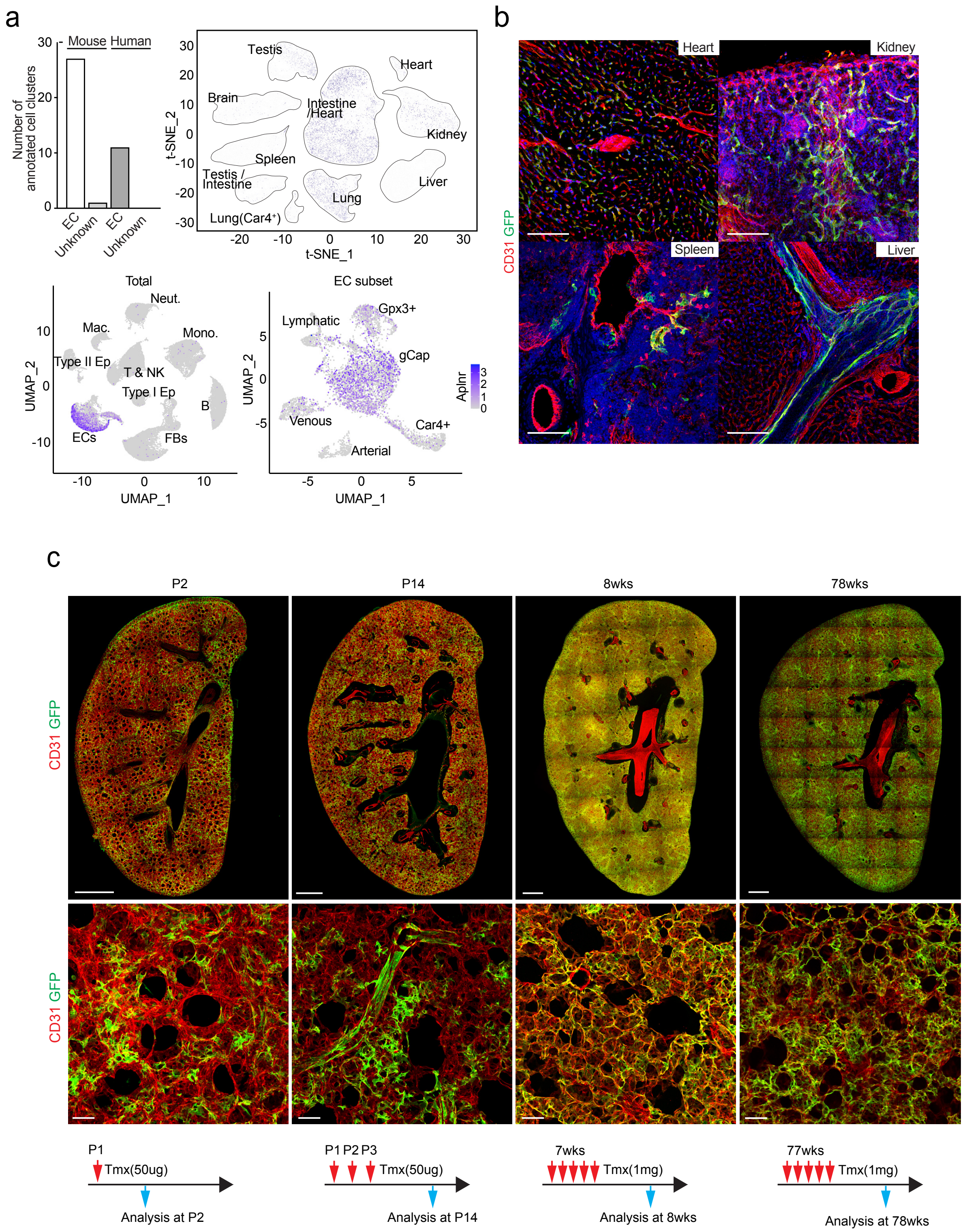

Supplementary Figure 4. Organ-specific differences in Aplnr expression.

a, Apelin receptor transcript expression in single-cell RNA-seq data. APLNR (human) or Aplnr (murine) expression is mostly confined to ECs according to different scRNA-seq databases (https://panglaodb.se/) (upper left). Aplnr expression in different adult organs according to the endothelial cell atlas[24] (https://endotheliomics.shinyapps.io/ec_atlas/) (upper right). Aplnr expression in lung scRNA-seq data from the current study (bottom left) and in the endothelial cell subset (bottom right). b, Confocal image of GFP expression (green) in the indicated Aplnr-CreERT2 R26i-mTmG adult organs after treatment with tamoxifen. ECs, CD31 (red). c, Images shown GFP+ cells in Aplnr-CreERT2 R26i-mTmG lung sections.Tamoxifen administration schemes and time of tissue analysis are indicated at the bottom. ECs, CD31 (red). Scale bar, 1 mm (upper panels), 50 µm (lower row). c, Images shown GFP+ cells in Aplnr-CreERT2 R26i-mTmG lung sections.Tamoxifen administration schemes and time of tissue analysis are indicated at the bottom. ECs, CD31 (red). Scale bar, 1 mm (upper panels), 50 µm (lower row).

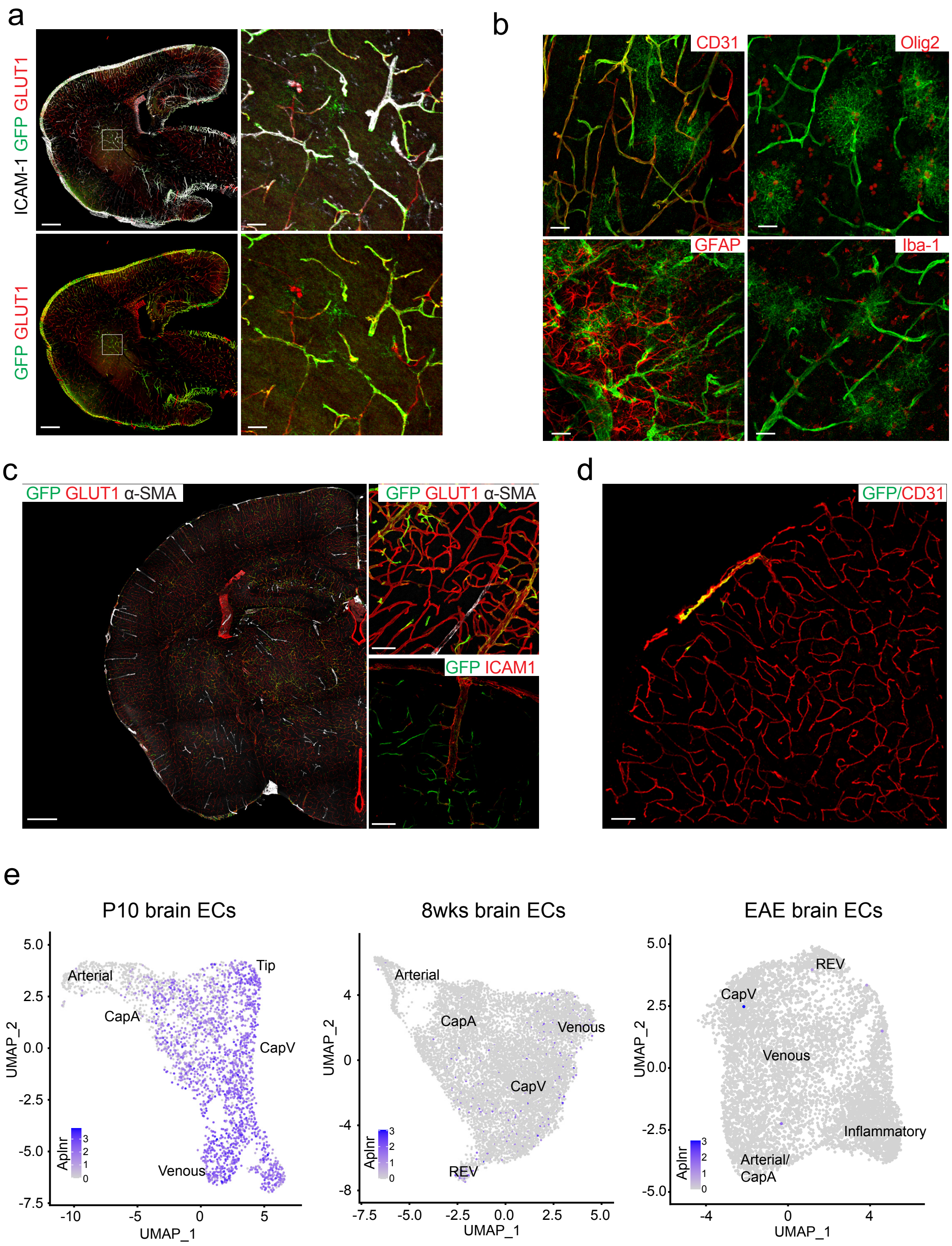

Supplementary Figure. 5: Aplnr expression in the brain vasculature.

a-d, Confocal images showing Aplnr-CreERT2 R26-mTmG sagittal brain sections. For acute labelling of Aplnr+ cells, tamoxifen was given at different developmental stages: P2 brain, with tamoxifen treatment at P1, immunostained for ICAM-1 (white) and GLUT1 (red) (a). P14 brain from mice treated with tamoxifen at P1-P3 and stained individually for CD31, Olig2, GFAP, and Iba-1 (b). P13 brains after treatment with tamoxifen at P12. Sections were stained for CD31 (red), SMA (white), and ICAM-1 (red), as indicated (c). GFP signal (green) is largely absent in 8-week-old brain treated with tamoxifen at 7 weeks. ECs, (red). (d). Scale bars, 200µm (left panels in a), 20µm (right panels in a), 10µm (b), 500µm (left panel in c), 100µm (right panelsin c), 50µm (d). e, UMAP plots of EC subsets in scRNA-seq data from naïve P10 and 8-week-old brain cortex as well as adult EAE brain cortex.

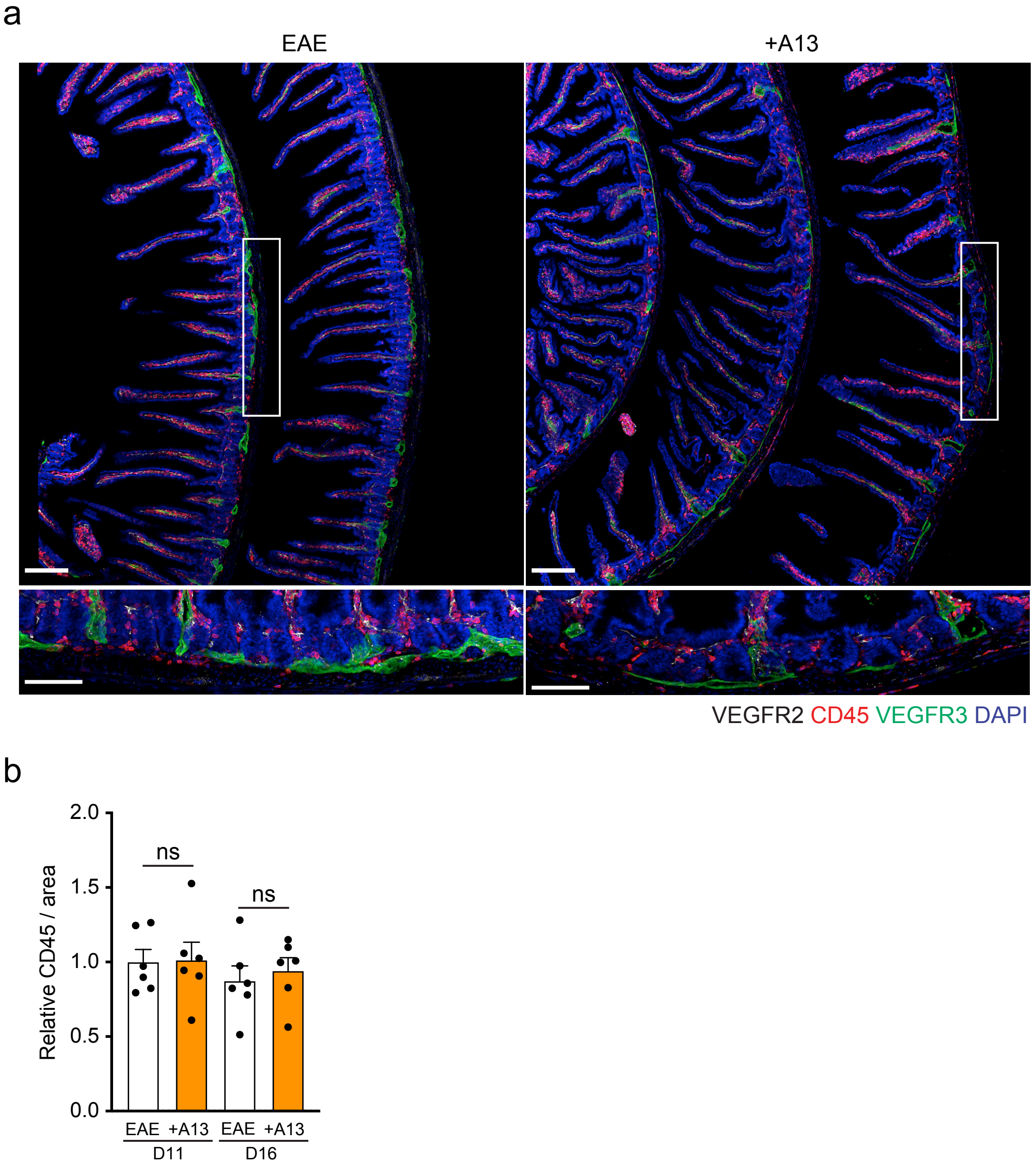

Supplementary Figure 6. A13 does not alter CD45+ cells in intestinal villi.

a, Confocal image of sectioned EAE and +A13 intestine at D16, stained for KDR/VEGFR2 (white), FLT4/VEGFR3 (green), CD45 (red) and DAPI (blue). Images in lower row show higher magnification of the indicated areas of the intestinal wall. Scale bars, 200µm (upper panels) and 100µm (lower panels). b, Quantification of CD45+ per area at D11 and D16. Error bars, s.e.m., Student t- test (ns, not statistically significant).). The number of mice is 6 for each group.

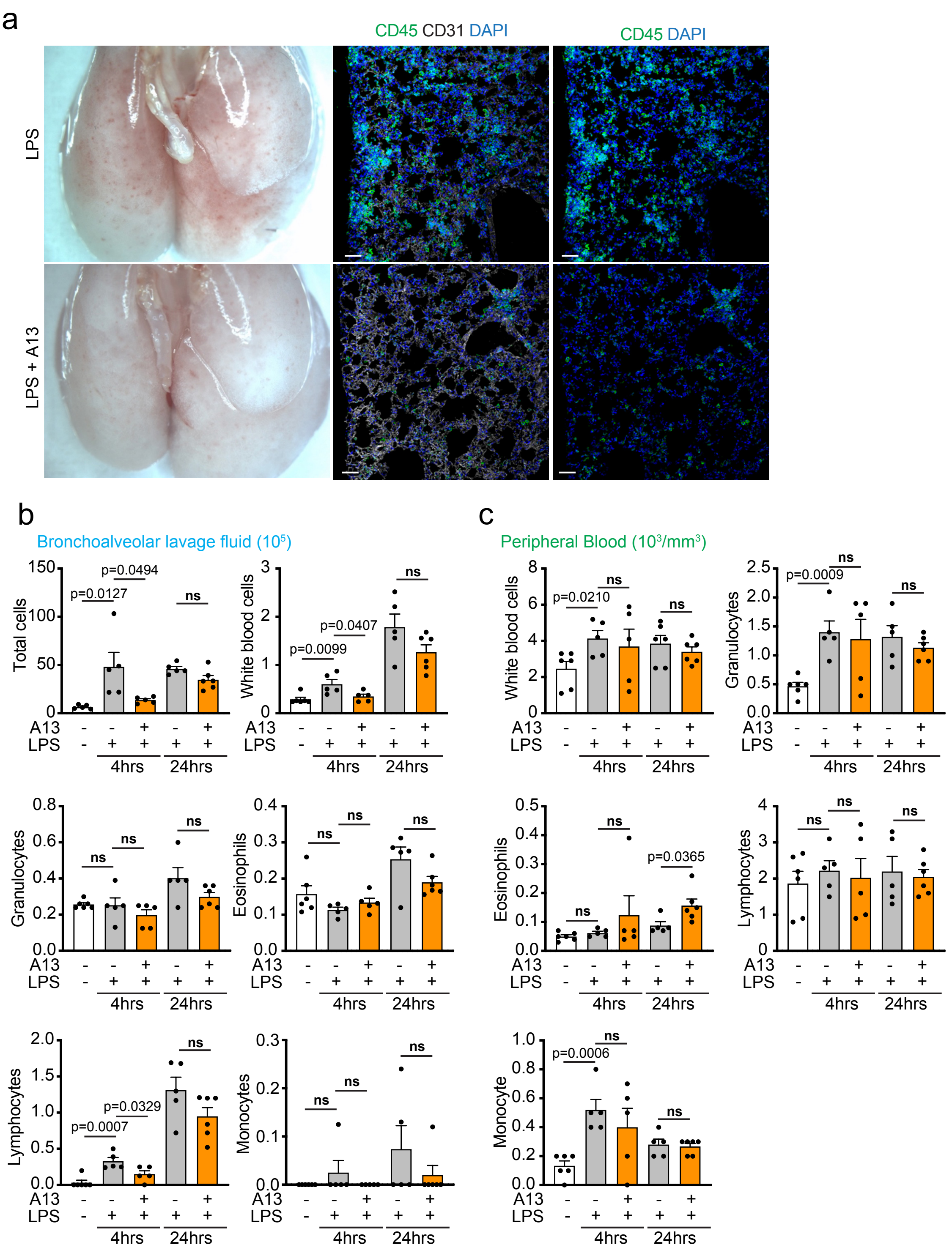

Supplementary Figure 7. A13 reduces LPS-induced lung inflammation.

a, Freshly isolated whole lungs and confocal images of lung sections stained for CD45 (green), CD31 (white), and with DAPI (blue) 18hrs after intranasal LPS administration. Scale bar, 50μm. b, c, The number of white blood cells and hematopoietic cell subpopulations in bronchoalveolar lavage fluid (b) and peripheral blood (c) at 4hrs and 24hrs after intranasal administration of LPS with or without A13 treatment, as indicated. Error bars, s.e.m. Student t-test (ns, not statistically significant). The number of mice is 5 for each group, except for the Naïve and 24hr A13 treated, which has 6 mice each.

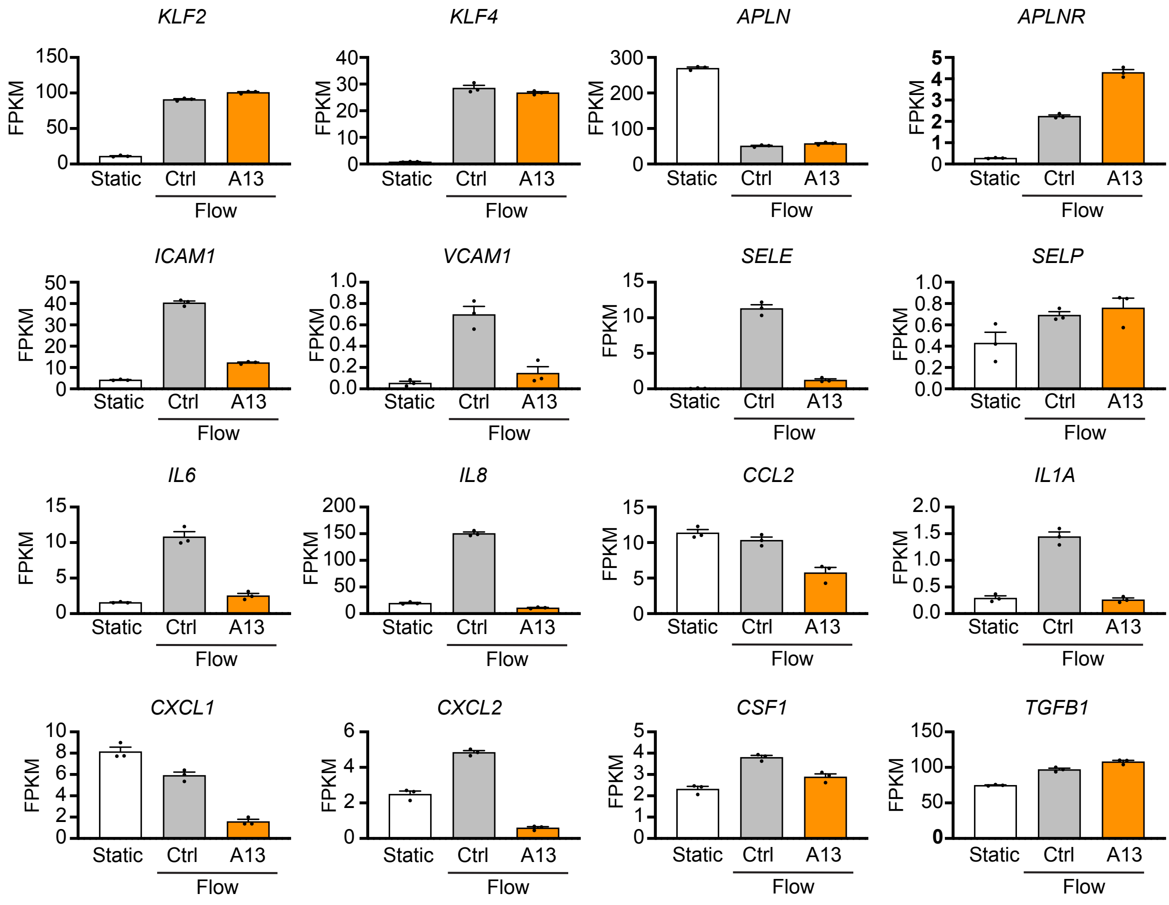

Supplementary Figure 8. Impact of flow and A13 on the expression of selected genes.

Analysis of the indicated gene transcripts in cultured HUVECs under static conditions, flow (15 dyn/cm^2 for 18 hours) or flow +Apelin-13 (A13) based on bulk RNA sequencing. Expression is shown as Fragments Per Kilobase of transcript per Million mapped reads (FPKM). Error bars, s.e.m. The number of samples is 3 for each group.

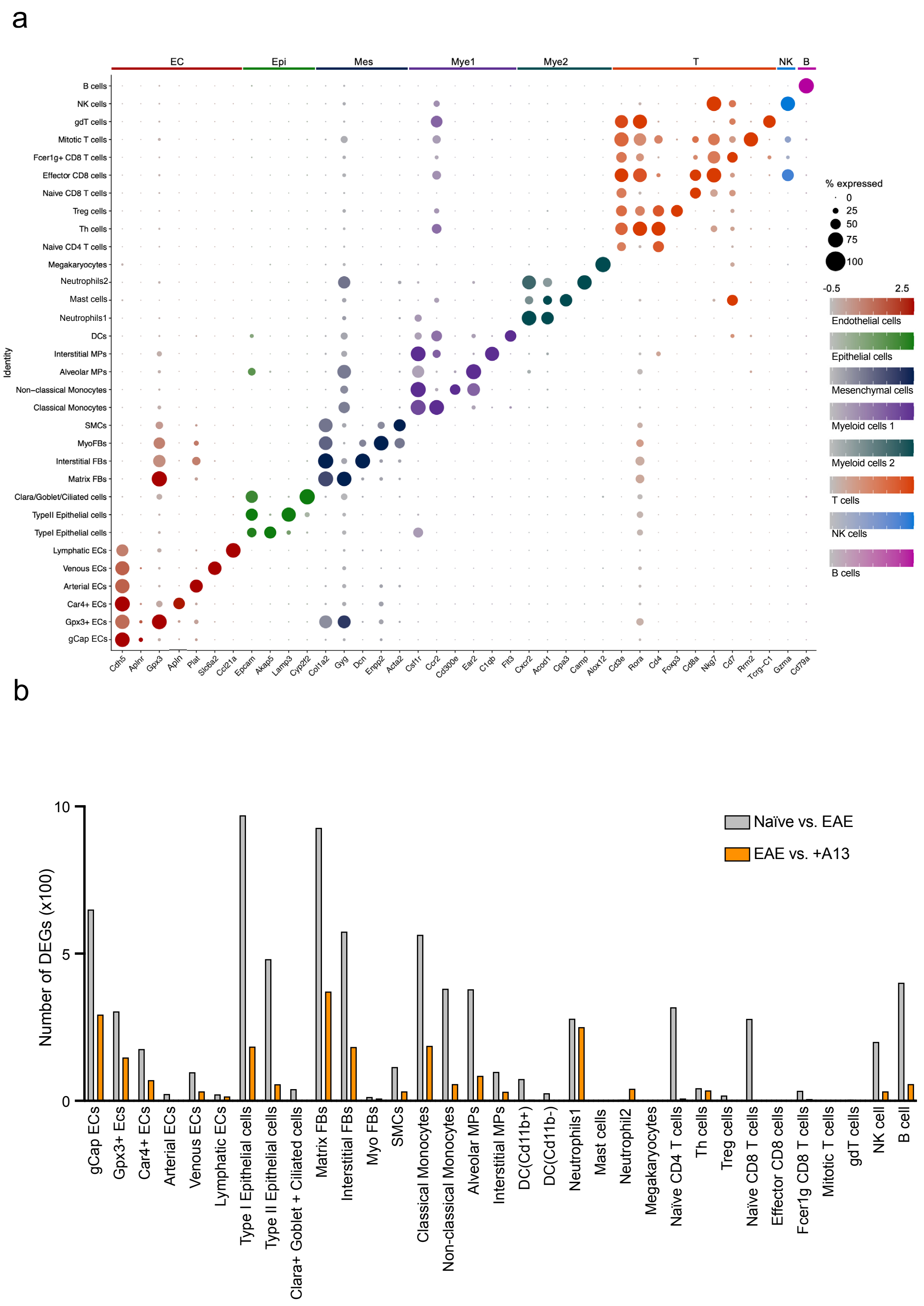

Supplementary Figure 9. Expression changes in different cell populations in vivo.

a, Expression of cell type-specific markers in lung scRNA-seq cell clusters. Color intensity represents the average expression level and dot size indicates the percentage of expressing cells. b, Number of differentially expressed genes (DEGs) (adjusted p-value<0.05, log2foldchang>0.25) for each subcell type. Comparison of EAE with naïve or +A13 with EAE is shown, as indicated.

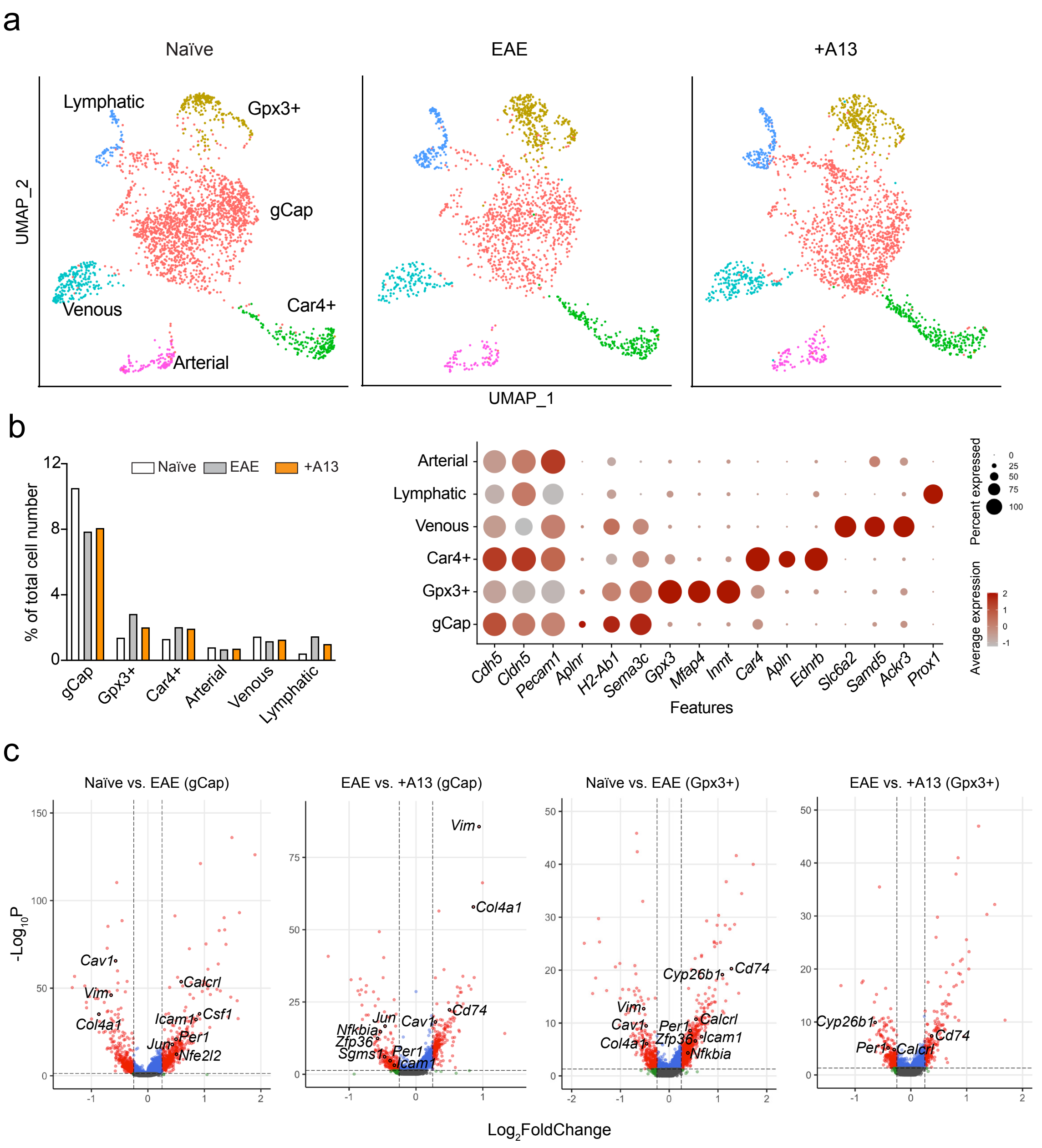

Supplementary Figure 10. Lung EC subset analysis by scRNA-sequencing.

a, UMAP plots of EC subsets from naïve, EAE, and EAE +A13 lungs. b, Percentage of total cell number represented by EC subclusters in the 3 conditions along with dot plot showing marker expression of EC subclusters. c, Volcano plots of gCap and Gpx3+ ECs comparing naïve with EAE or EAE with EAE +A13 conditions.

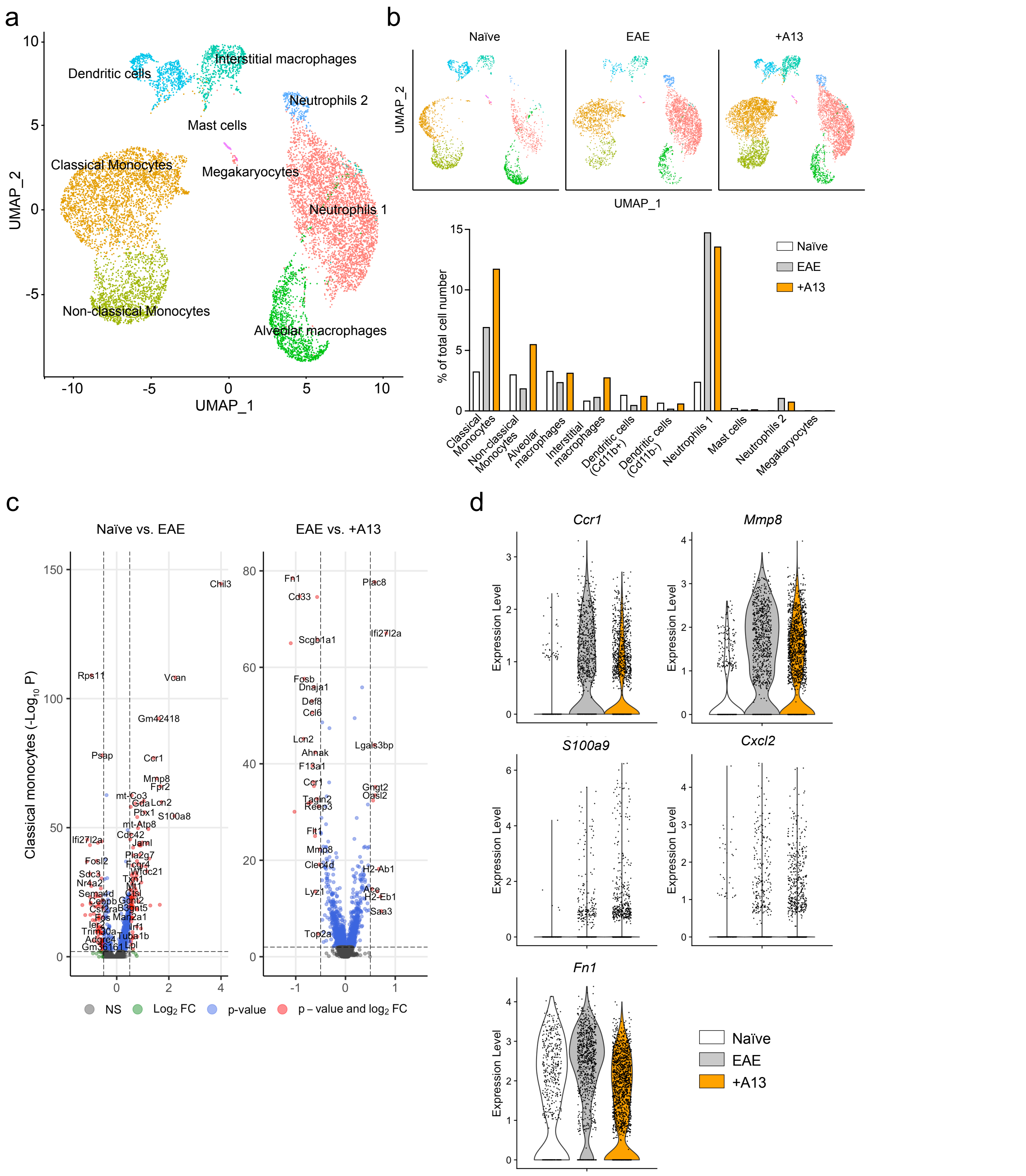

Supplementary Figure 11. Myeloid subset analysis lung by single-cell RNA sequencing.

a, UMAP plot of the myeloid cell subset. b, UMAP plots of myeloid cell subclusters separated by conditions (top panels) along with the percentage of total cell number in each subcluster (bottom). c, Volcano plots comparing classical monocytes between Naïve and EAE as well as EAE and EAE +A13. d, Violin plots showing the expression distribution of selected genes in classical monocytes.

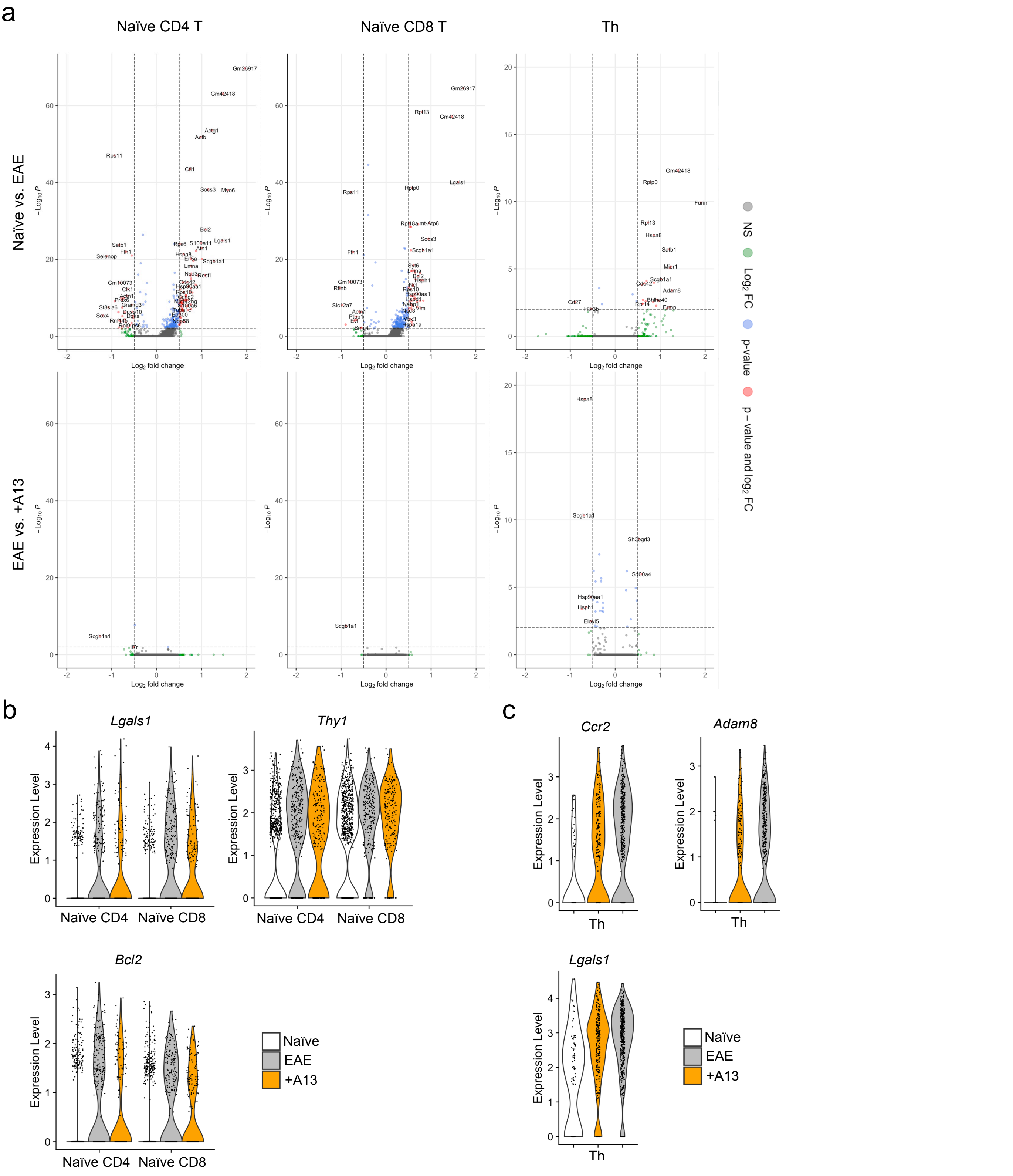

Supplementary Figure 12. Gene expression analysis of T lymphocytes.

a, Volcano plots comparing CD4 T cells, CD8 T cells, and Th cells from lung between naïve and EAE as well as EAE and +A13 conditions. b, Violin plots showing the expression of Lgals1, Thy1, and Bcl2 in CD4 and CD8 T cells from lung in different conditions. c, Violin plots showing the expression of Ccr2, Adam8, and Lgals1 in Th cells.

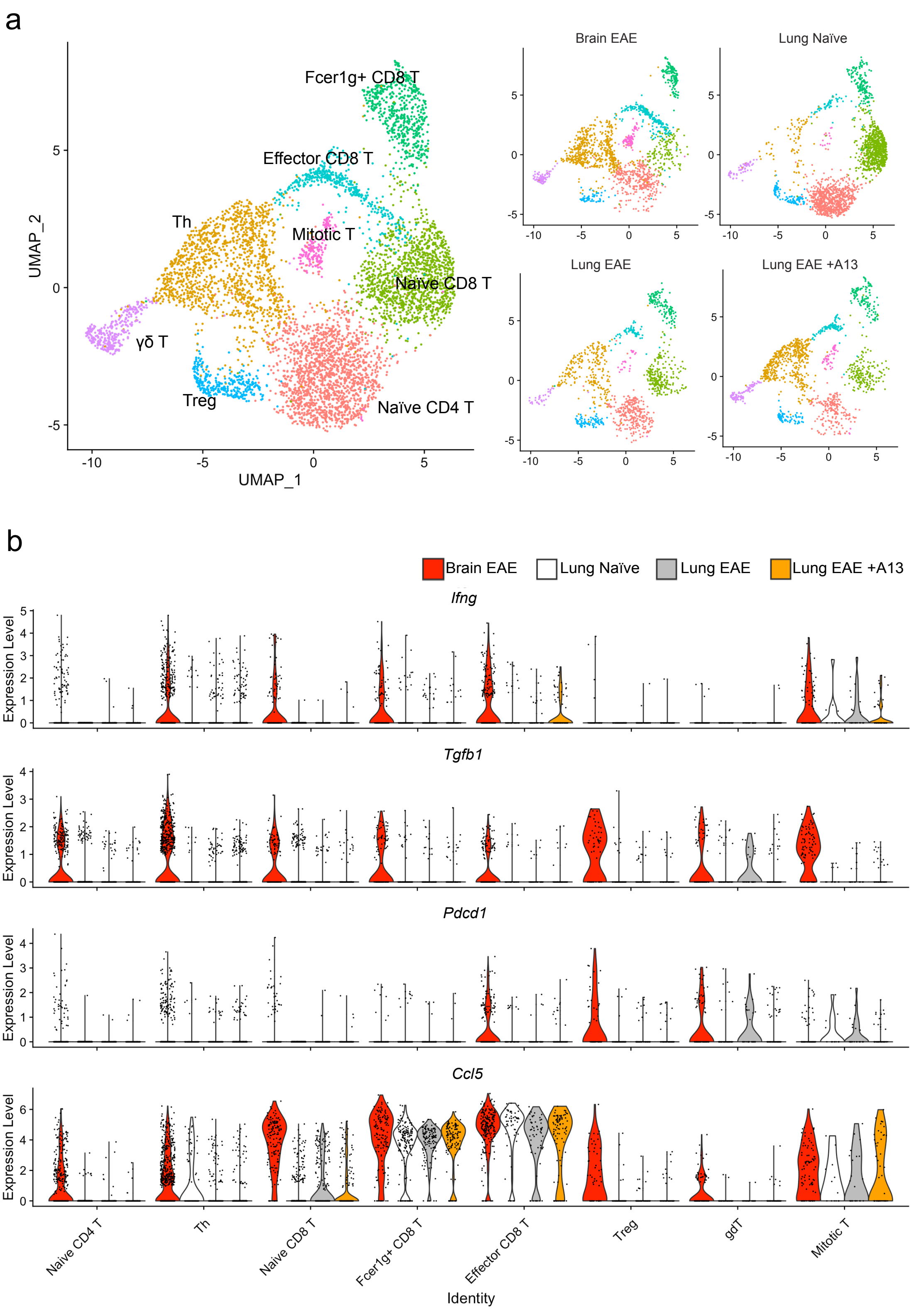

Supplementary Figure 13. Analysis of T cell scRNA-seq data from brain and lung.

a, UMAP plots showing the distribution of total T cells and samples from individual conditions, namely EAE brain, naïve lung, EAE lung, and EAE +A13 lung. b, Violin plots depicting the expression of Ifng, Tgfb1, Pdcd1, and Ccl5 in T cell subclusters for the different conditions. Note high expression of Ifng, Tgfb1 and Pdcd1 in T cells from EAE brain relative to lung samples.

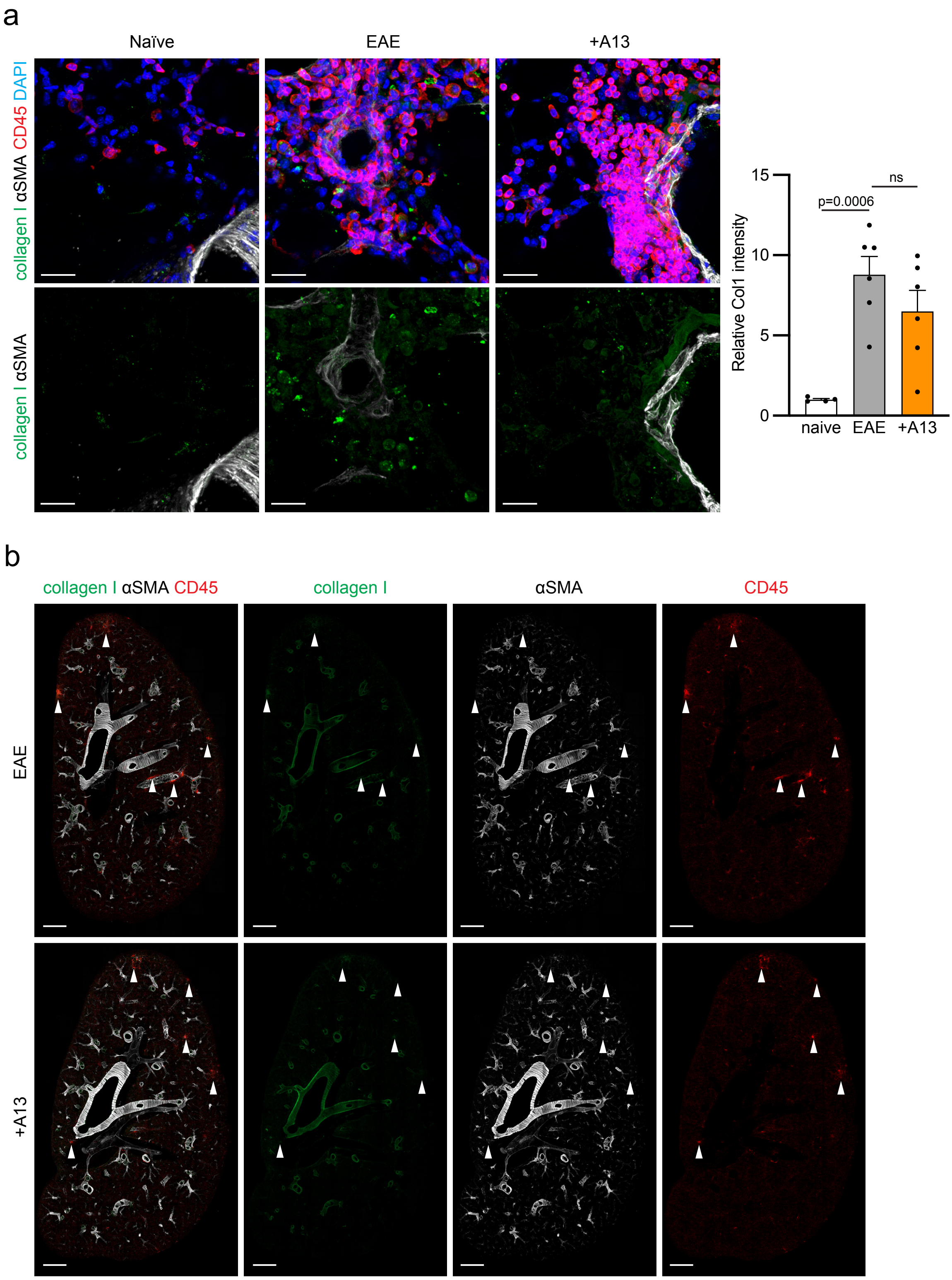

Supplementary Figure 14. No indication of lung fibrosis due to A13-induced CD45+ cell accumulation.

a, Lung sections from naïve, EAE, and EAE +A13 (D16) stained for collagen type I (green), αSMA (white), CD45 (red) and with DAPI (blue). Note absence of increased αSMA immunosignals in areas containing CD45+ cells. Quantitation of collagen type I intensity normalized by naïve lung. Error bars, s.e.m. Student t-test (ns, not statistically significant), Scale bar, 30µm. The number of mice is 6 for each group, except for the Naïve, which has 4 mice. b, Representative confocal image of sectioned EAE and +A13 lung lobe at D30 stained with collagen type I (green), αSMA (white), and CD45 (red). Scale bar, 1 mm.

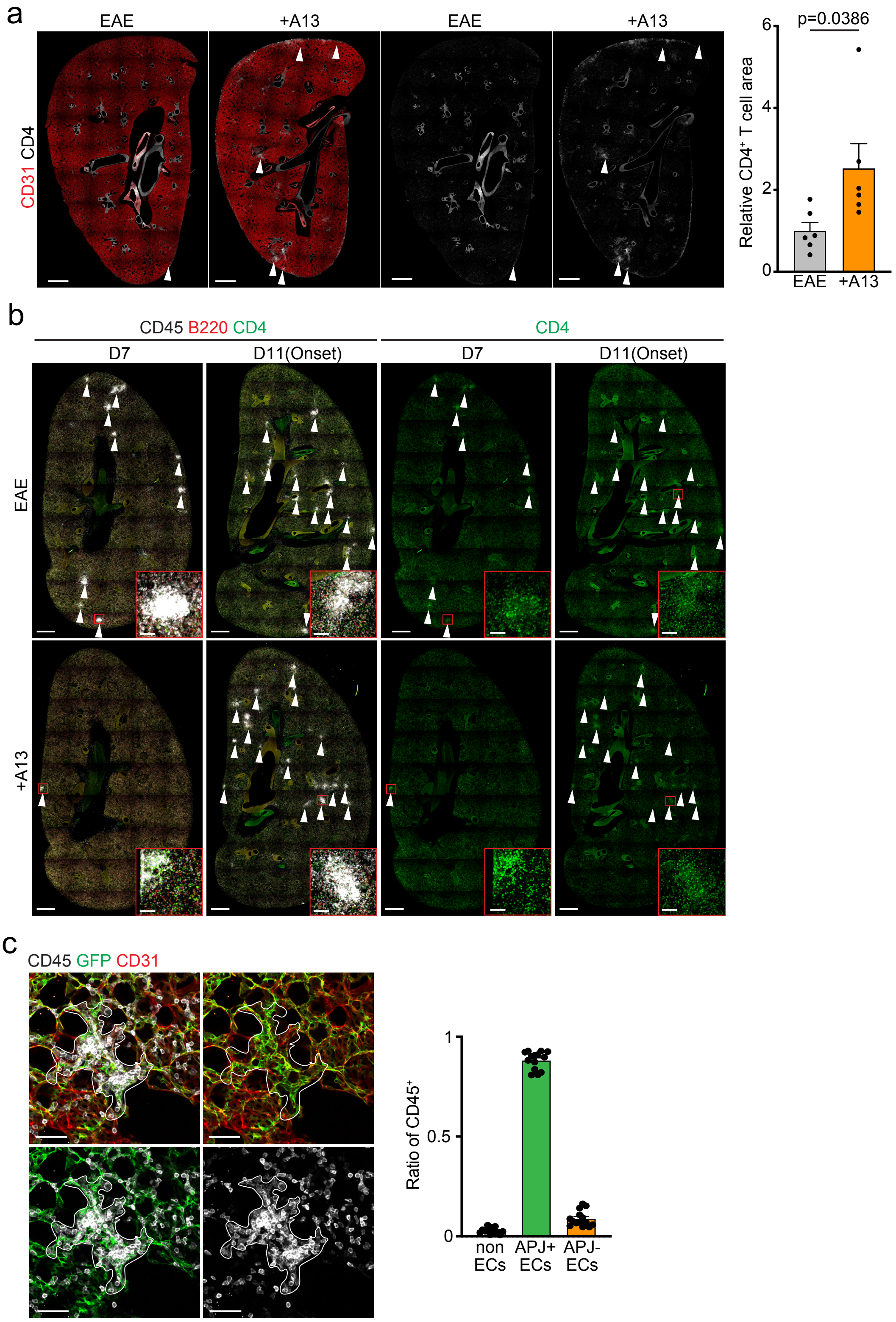

Supplementary Figure 15. A13 delays T cell accumulation in lung during EAE.

a, Confocal images of lung sections and corresponding quantitation from EAE and +A13 conditions (at D16), immunostained for CD31 (red) and CD4 (white). White arrowheads indicate immune cell clusters. Scale bar, 1 mm. Error bars, s.e.m. Student t-test. The number of mice is 6 for each group. b, EAE and +A13 lung sections taken at D7 or D11 stained for CD45 (white), B220 (red), and CD4 (green). White arrowheads indicate immune cell clusters. Note that A13 causes an initial delay in immune cell cluster formation. Scale bar, 1mm. c, Confocal images of CD45+ (white) immune cell cluster (white outline) in tamoxifen- treated Aplnr-CreERT2 R26i-mTmG lungs stained for GFP (green) and CD31 (red). Scale bar, 100 µm. Graph on the right shows ratio of CD45+ cells in proximity of GFP+ (Aplnr/APJ+) ECs, APJ- ECs or without contact to ECs (no ECs). 14 areas from 3 mice for EC GFP+ (Aplnr/APJ+) analysis (as shown in c).

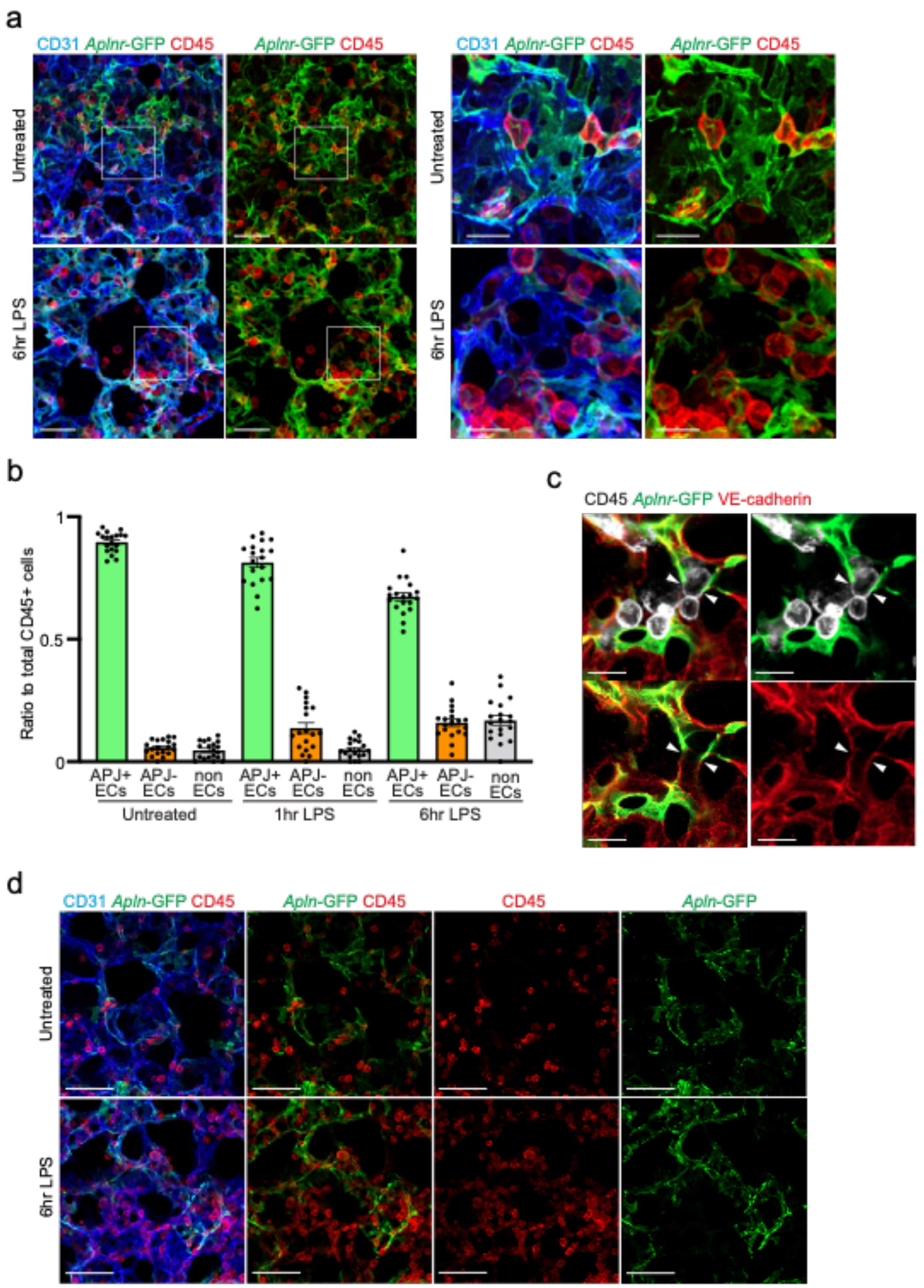

Supplementary Figure 16. Immune cells and Aplnr^{+} ECs in LPS-treated lungs.

a, Confocal images of lung sections from LPS-treated Aplnr-CreERT2 R26-mTmG mice stained for CD31 (blue), CD45 (red) and GFP (green). Scale bar, 30µm. Panels on the right show higher agnification of insets. Scale bar, 10µm. b, Quantitation of immune cell contact with GFP+ (Aplnr+) CD31+ ECs and GFP- CD31+ ECs or without CD31 cell contact in lung or after intranasal LPS administration. Error bars, s.e.m. 19 images from 3 mice for each conditions. c, High-magnification images of Aplnr-CreERT2 R26-mTmG lung sections exposed to EAE. Immunostaining shows CD45 (white), VE-cadherin (red) and GFP (green). White arrowhead indicate immune cell transmigration through Aplnr+ ECs. d, Confocal image of lung sections from LPS-treated Aplnr-CreERT2 R26-mTmG lung and untreated control stained for CD31 (blue), CD45 (red) and GFP (green). Scale bar, 50µm.

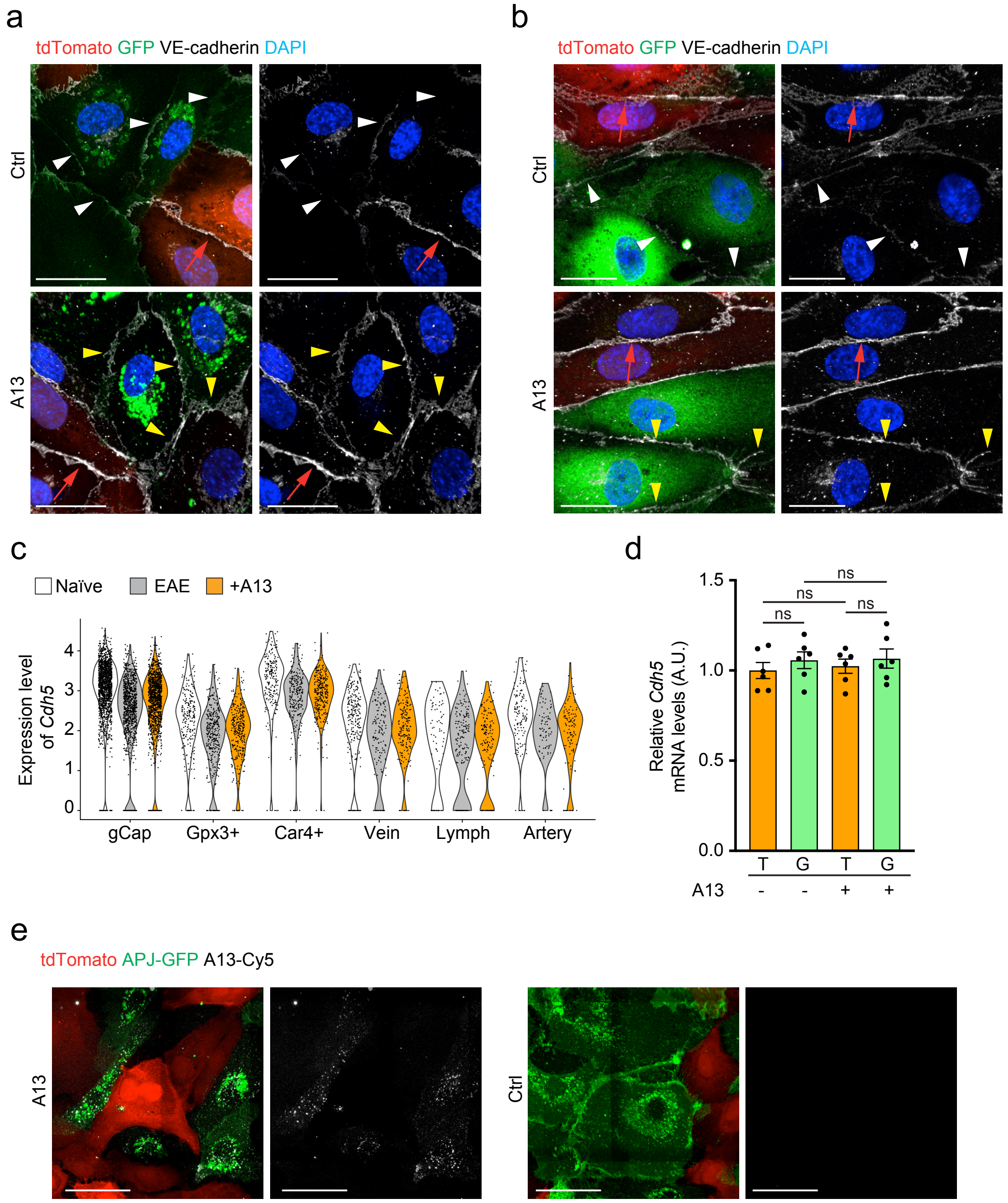

Supplementary Figure 17. Effect of APJ overexpression and A13 on junctional VE- cadherin.

a, Confocal images of co-cultured HUVECs expressing GFP-tagged APJ (APJ-GFP, green) or tdTomato (red), with or without A13 treatment. Cells were stained with antibody against VE-cadherin (white) and DAPI (blue). Junctional VE-cadherin of APJ-GFP+ HUVECs before (white arrowheads) and after A13 treatment (yellow arrowheads) is indicated. Red arrows mark junctions between tdTomato+ cells. Scale bar, 20 µm. b, Confocal images of co-cultured HUVECs expressing APJ-T2A-GFP or tdTomato, with or without A13 treatment. Cells were stained with antibodies binding VE-cadherin (white) and DAPI (blue). Junctional VE-cadherin of APJ-T2A-GFP+ HUVECs before (white arrowheads) and after A13 treatment (yellow arrowheads) is indicated. Red arrows mark junctions between tdTomato+ cells. Scale bar, 20 µm. c, Cdh5 expression in scRNA-seq data of lung EC subsets from Naïve, EAE (vehicle-treated control), and EAE +A13 conditions. d, RT-qPCR analysis of Cdh5 expression in APJ-GFP or tdTomato HUVECs with or without A13 treatment. Error bars, s.e.m. Student t-test (ns, not statistically significant), Duplication of 3 samples (total 6) for each conditions. e, Confocal images of co-cultured APJ-GFP (green) and tdTomato-expressing (red) HUVECs with or without treatment with Cy5-tagged A13 (A13-Cy5, white). Scale bar, 50µm.

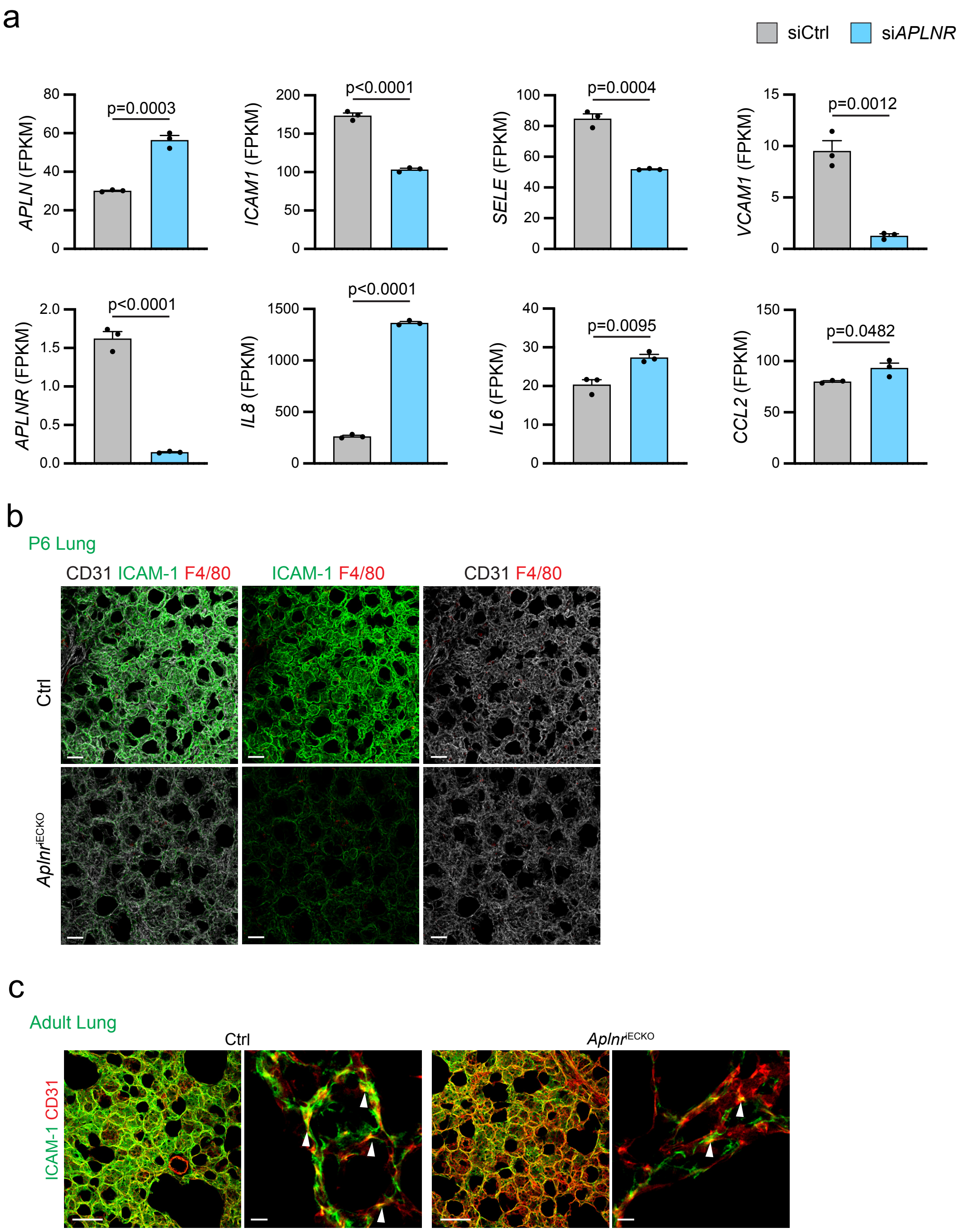

Supplementary Figure 18. Loss of Aplnr reduces ICAM-1 expression in ECs.

a, Bulk RNA-seq analysis (FPKM values) of selected genes from siCont or siAPLNR treated HUVECs under flow (*p<0.05, **p<0.01, ***p<0.001,****p<0.0001), The number of samples is 3 for each group. b, P6 lung of Aplnr^{iECKO} and littermate control after treatment with tamoxifen at P1-3 and staining for CD31 (white), ICAM-1 (green), and F4/80 (red). Scale bar, 100µm. c, Confocal images of lung sections from adult Aplnr^{iECKO} mutants and littermate controls at D16 stained for ICAM-1 (green) and CD31 (red). Arrowheads indicate endothelial ICAM-1 and downregulation in Aplnr^{iECKO} lung. Scale bar, 100µm (left) and 10µm (right).

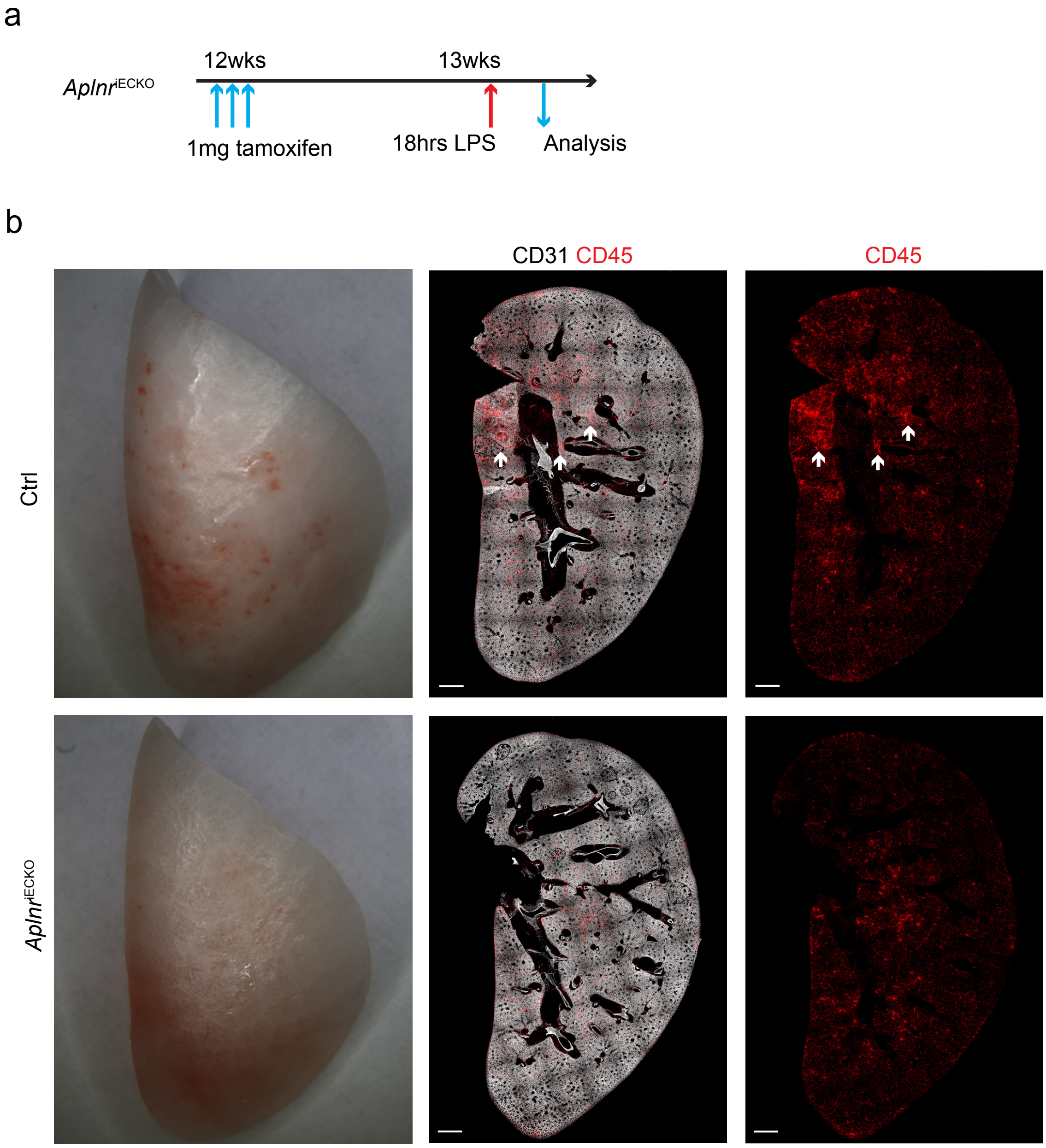

Supplementary Figure 19. LPS-induced lung inflammation in Aplnr^{iECKO} mutants.

a, 12-week-old female and sex-matched littermate controls were treated with tamoxifen for 3 days and subsequently challenged with LPS for 18 hours at 13 weeks. b, Freshly isolated lung lobes and confocal images of section stained for CD31 (white) and CD45 (red). Note reduction of CD45+ clusters (arrowheads) in Aplnr^{iECKO} lung. Scale bar, 1 mm.

a

b

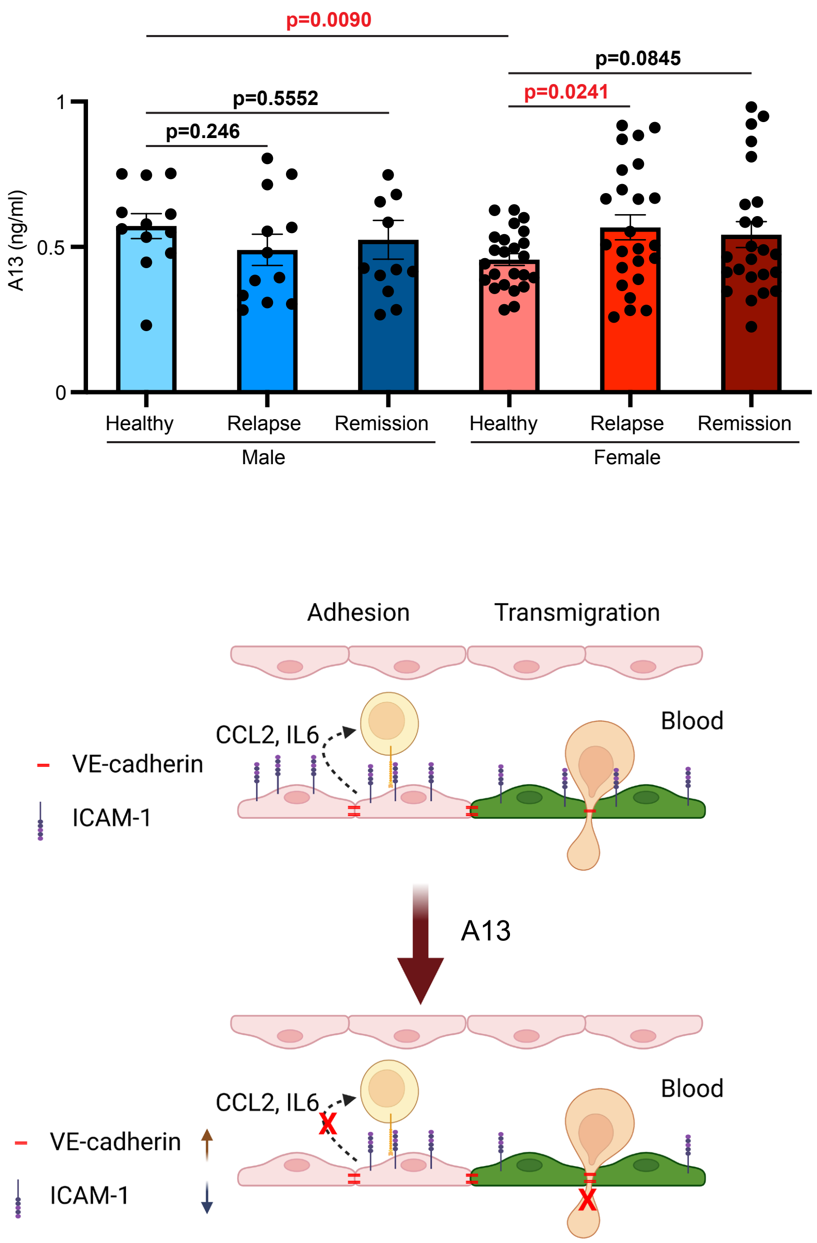

Adapted from “Alveolar-Capillary Barrier (Background)”, by BioRender.com (2023). Retrieved from https://app.biorender.com/biorender-templates

Supplementary Figure 20. Role of A13 in immune cell recruitment.

a, A13 levels in human serum measured by Elisa. Samples were taken from male (12 people for each condition) or female (24 people for each condition) healthy donors, MS patients in relapse, and MS patients in remission. Patient Metadata is provided as Supplementary Dataset 1. Statistical significance is indicated by red p-values (student t-test). b, Schematic diagram showing the effect of A13 on endothelial cells and the resulting impairment of immune cell adhesion and transendothelial migration.

FACS gating strategy

a, For EC isolation

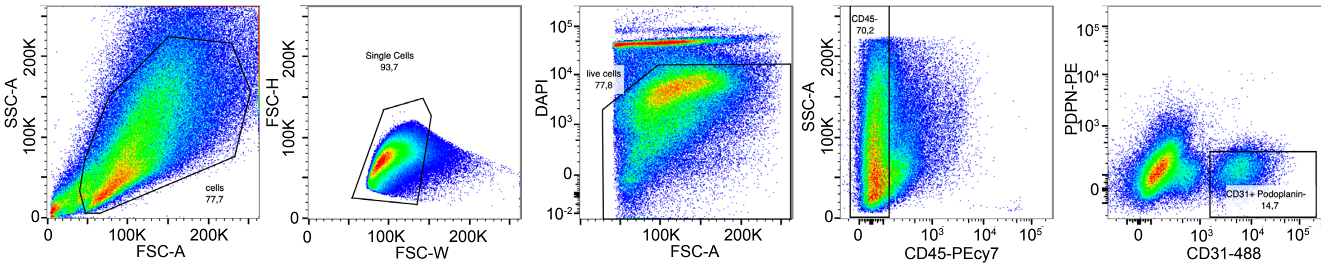

b, For CD45, CD4 and CD8 T cell analysis in brain (before activation for Th cell)

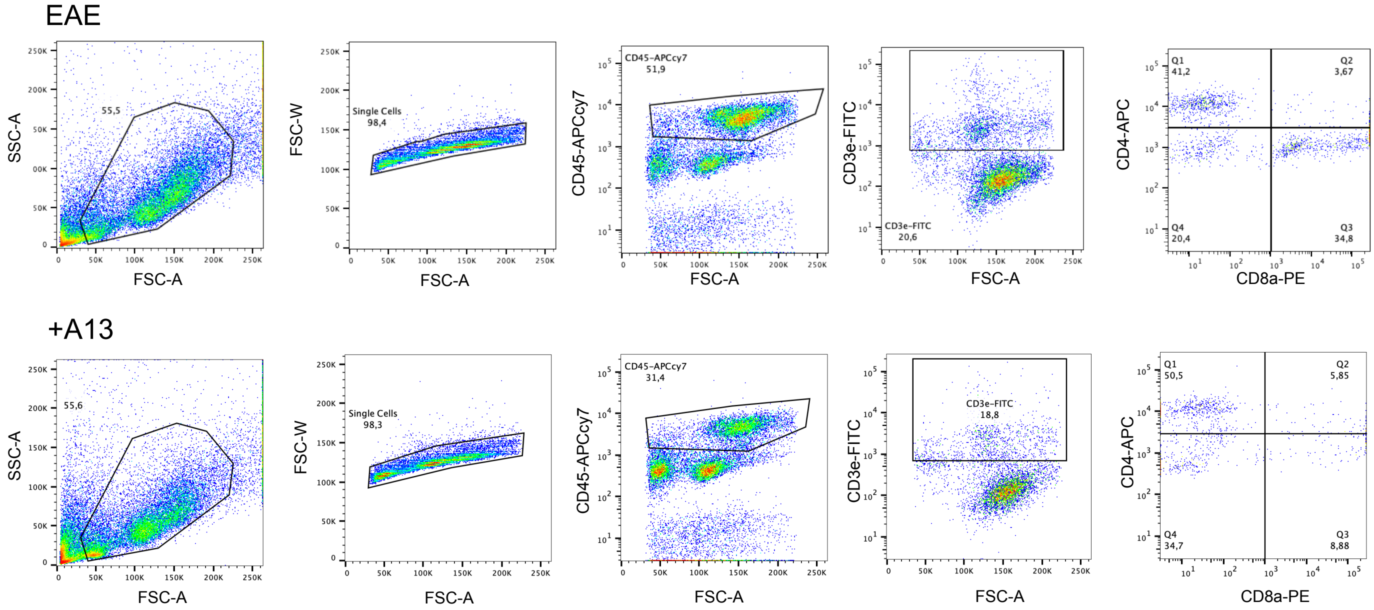

c, For CD4 Th1 and Th17 cell analysis in brain (after activation for Th cell)

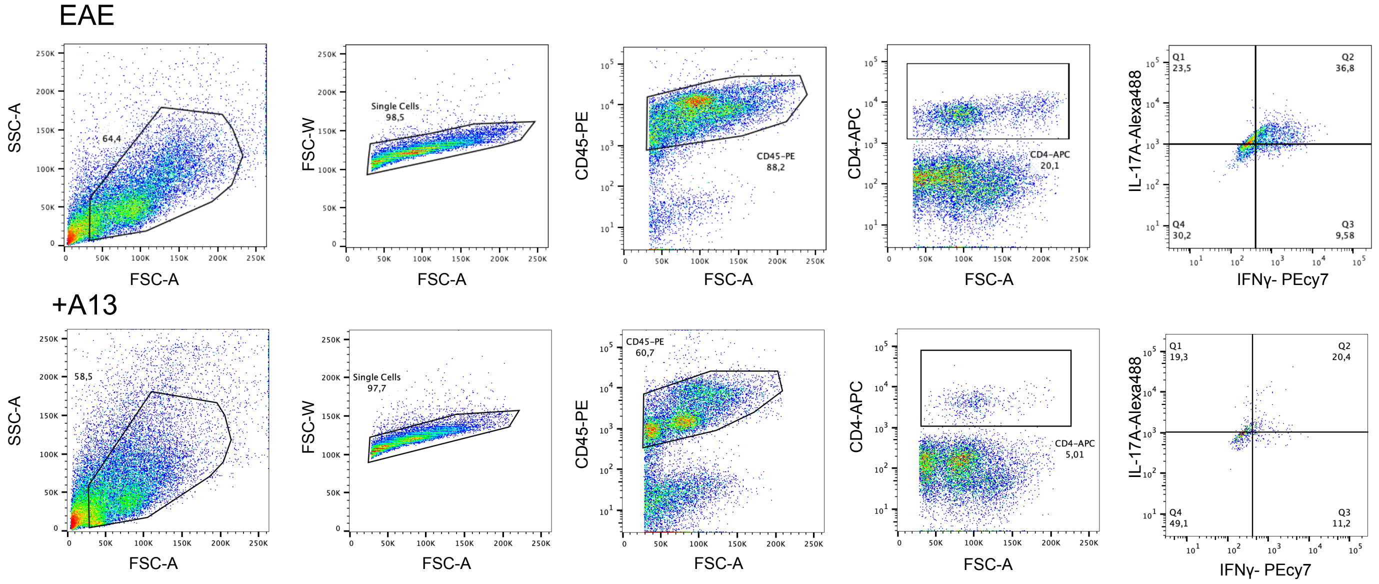

a, For CD45, CD4 and CD8 T cell analysis in lung (before activation for Th cell)

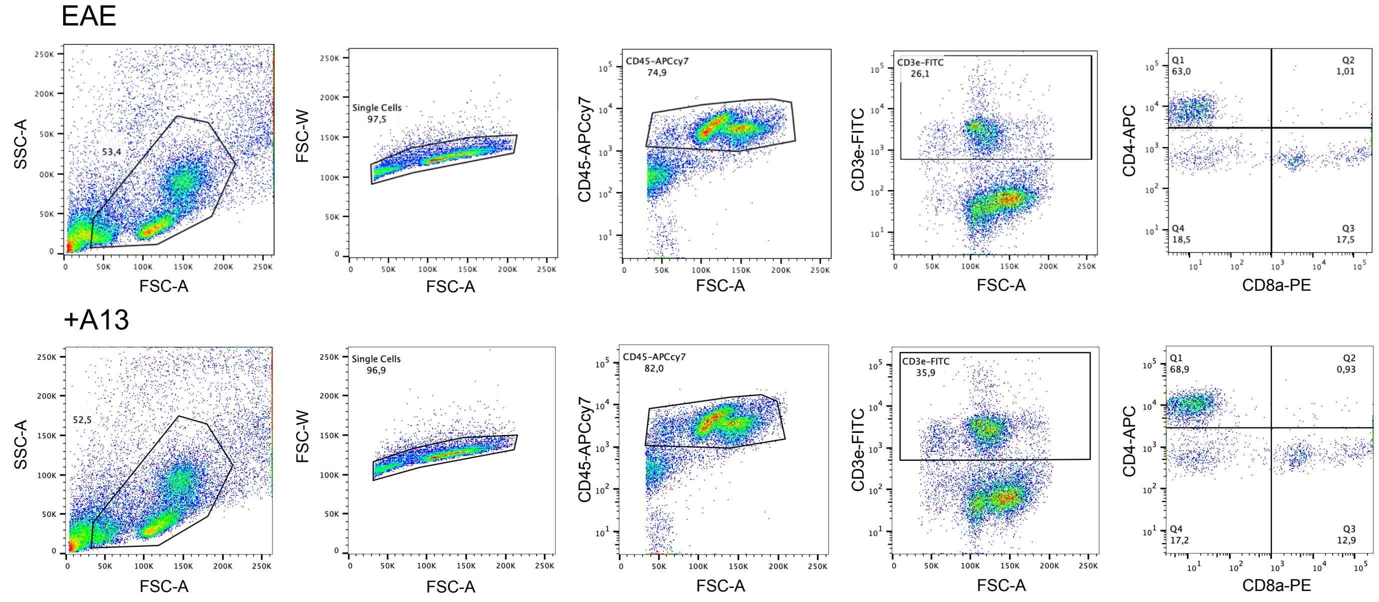

b, For CD4 Th1 and Th17 cell analysis in lung (after activation for Th cell)

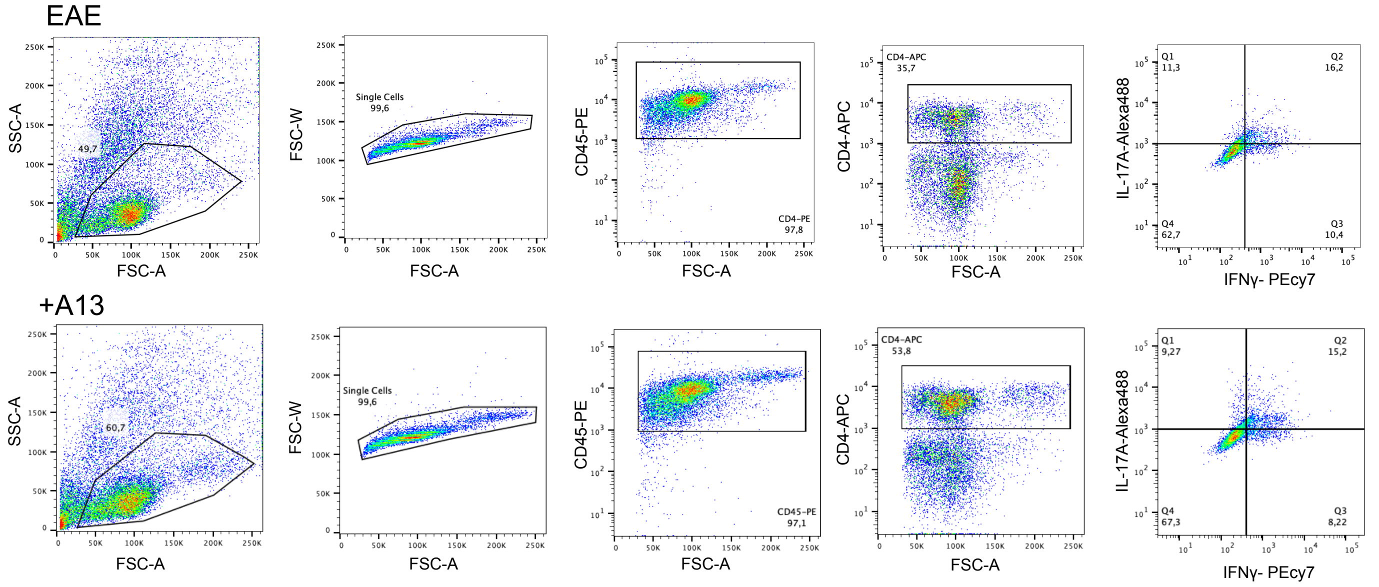

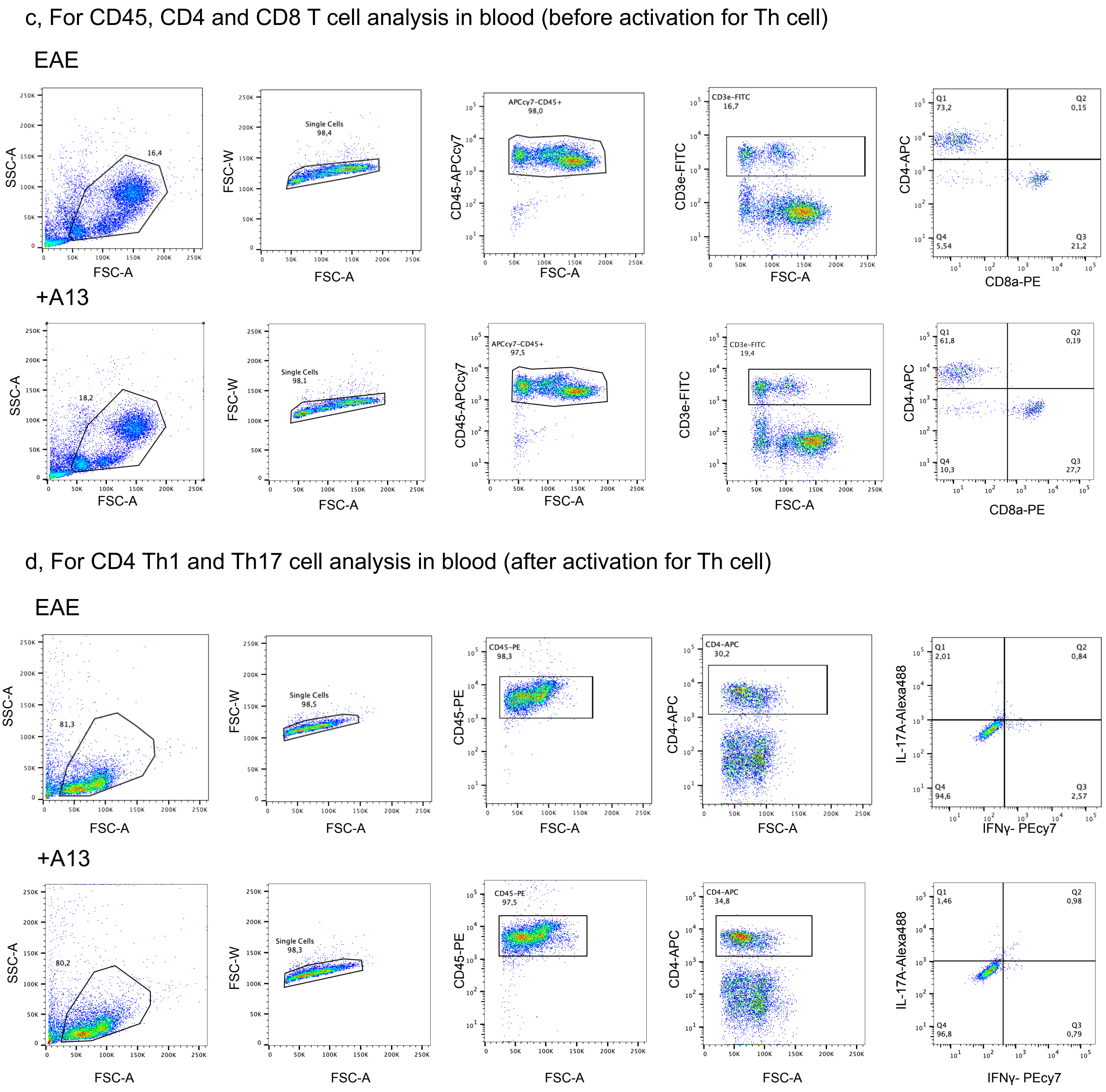

Supplementary Figures 21. Representative FACS plots showing gating strategies.

FACS for Th1 and Th17 required stimulation, fixation and permeabilization of cells.

Figure 5D

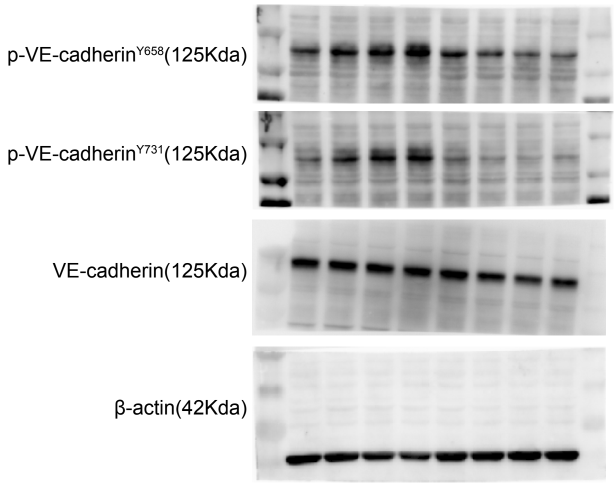

Supplementary Figure 22. Uncropped Western blots.

Uncropped Western blots for data in Fig. 5d.
