# Supplementary material for: Apelin modulates inflammation and leukocyte recruitment in experimental autoimmune encephalomyelitis
Source: Nat Commun. 2024 Jul 25;15:6282. doi: 10.1038/s41467-024-50540-5 (PMC11282314; doi:10.1038/s41467-024-50540-5)
Supplement: Supplementary file 3 — Description of Additional Supplementary Files [file 41467_2024_50540_MOESM3_ESM.pdf]

Description of Additional Supplementary Files

File Name: Supplementary Data 1

Description: Patient information for A13 levels in human serum Elisa experiment in Supplementary Figure 20a (Age, Sex, MS Type, Diagnosis, Therapy).

File Name: Supplementary Movie 1

Description: tdTomato HUVEC and T cells.

File Name: Supplementary Movie 2

Description: APJ-GFP HUVEC and T cells.

File Name: Supplementary Movie 3

Description: tdTomato HUVEC and T cells treated with A13.

File Name: Supplementary Movie 4

Description: APJ-GFP HUVEC and T cells treated

with A13.
